# Comparison of in-house IgM and IgG ELISAs for the serodiagnosis of melioidosis in Malaysia

**DOI:** 10.1099/jmm.0.000611

**Published:** 2017-10-19

**Authors:** Shirley Yi Fen Hii, Noor Azila Ali, Norazah Ahmad, Fairuz Amran

**Affiliations:** Bacteriology Unit, Infectious Diseases Research Centre, Institute for Medical Research, 50588 Jalan Pahang, Kuala Lumpur, Malaysia

**Keywords:** melioidosis, in-house ELISA, Malaysia, IgM, IgG

## Abstract

Melioidosis is an endemic infectious disease in Southeast Asia and northern Australia, caused by *Burkholderia pseudomallei*. However, the incidence rate in Malaysia is not well documented. The high mortality rate and broad range of clinical presentations require rapid and accurate diagnosis for appropriate treatment. This study compared the efficacy of in-house IgM and IgG ELISA methods using a local *B*. *pseudomallei* strain. The diagnostic accuracy of the in-house IgG ELISA was better than that of the IgM ELISA: sensitivity (IgG: 84.71 %, IgM: 76.14 %) and specificity (IgG: 93.64 %, IgM: 90.17 %); positive predictive value (IgG: 86.75 %, IgM: 79.76 %) and negative predictive value (IgG: 92.57 %, IgM: 89.66 %); likelihood ratio (LR) [IgG: 13.32, IgM: 7.75 (LR+); IgG: 0.16, IgM: 0.26 (LR–)], and was supported by the observation of the absorbance value in comparisons between culture and serology sampling. In-house IgG ELISA was shown to be useful as an early diagnostic tool for melioidosis.

## Introduction

Melioidosis is a potentially fatal disease caused by *Burkholderia pseudomallei* and is endemic to Southeast Asia and northern Australia. A recent report by Limmathurotsakul showed that the incidence of melioidosis was highly underestimated even in endemic countries [1]. The diagnosis of melioidosis is difficult due to its diverse clinical manifestations ranging from subclinical infection to acute fatal septicaemia [1–3]. To date, there is no vaccine available for melioidosis. Although effective antibiotics are available, melioidosis treatment requires prolonged antimicrobial therapy, and the outcome can be fatal if the disease is misdiagnosed [3, 4].

The gold standard for the diagnosis of melioidosis mainly depends on the traditional culture method [2, 3]. However, the isolation of *B. pseudomallei* from body fluids requires 3–5 days and in some cases the bacterium is not always isolated [3, 5]. Therefore, the use of a sensitive and specific serology test can be an alternative for the rapid diagnosis of melioidosis. Routine serological tests for the diagnosis of melioidosis include indirect haemagglutination (IHA), ELISA and immunofluorescent assay (IFAT) [5, 6]. Despite being applied worldwide, IHA was shown to be less accurate in endemic regions, as documented in Thailand where there was high seropositivity in healthy subjects [7], while IFAT requires a fluorescence microscope and skilled personnel to interpret the results [6].

Hence, the ELISA method, which is sensitive, specific, rapid, cost-effective and user-friendly, is favourable [8, 9]. The objective of this study was to compare the efficiency and accuracy of the in-house IgM and IgG ELISA methods using locally isolated *B. pseudomallei* and to determine the cut-off value for early diagnosis of melioidosis in Malaysian patients.

## Methods

### Human serum samples

This study was conducted using sera collected from January 2016 to June 2017. The sera were received by the Institute for Medical Research for routine serological diagnosis. The sera were stored at −20 °C prior to use. In this study, a selection of 258 serum samples which consisted of sera from 85 *B. pseudomallei* culture-positive cases and sera from 173 culture-negative cases, was used. Repeated samples were excluded in the study. Out of the 85 culture-confirmed sera (66 males, 19 females; age range 2 months to 84 years old), there were 64 septicaemic and 21 localized infections. The usual clinical presentations included fever, cough, abcesses and abdominal pain [3, 10]. The negative controls were healthy blood donors (*n*=108), and sera from patients diagnosed with *Legionella pneumonia* infection (*n*=11), *Burkholderia cepacia* infection (*n*=9), leptospirosis (*n*=20), brucellosis (*n*=10) and rickettsiosis (*n*=15).

### Bacterial strain

The *B. pseudomallei* B124E strain was obtained from the Institute for Medical Research Culture Collection Centre. This strain was previously isolated from a clinical specimen, multilocus sequence typed as ST 289 and characterized based on biochemical tests and PCR sequencing of the 16S rRNA region.

### Enzyme-linked immunosorbent assay

ELISA was performed in 96-well flat bottom immunoplates (SPL) as previously described with minor modifications [11]. The wells were coated with 75 µl *B. pseudomallei* whole cell antigen suspension in each well (absorbance: 0.10 at 620 nm). The antigens were initially heat-inactivated at 100 °C for 15 min before coating of wells. Upon drying, the wells were washed three times with PBS-T (PBS pH 7.2 plus 0.05 % Tween 20) and blocked with blocking buffer [3 % (w/v) skimmed milk in PBS] for 90 min at room temperature. After three washes with PBS-T, duplicate wells were incubated at 37 °C for 30 min with various titres of human sera samples (1 : 160 to 1 : 640) to a volume of 100 µl. After incubation, wells were washed three times with PBS-T before the addition of 100 µl peroxidase-labelled antibody to human IgM/IgG (KPL). Following three washes with PBS-T, nine volumes of substrate A [0.08 % (w/v) 5 aminosalicylic acid (MP Biomedicals)] was mixed with one volume of substrate B (0.05 % hydrogen peroxide) prior to addition of 100 µl mixture to each well. The reaction was left to develop for 1 h at 37 °C and read at 492 nm with an automated ELISA reader (Tecan).

### Determination of cut-off value

All measurements of the tested sera samples were done in duplicate. The mean and standard deviations were calculated. The cut-off value was determined by choosing the highest proportion of correctly classified patients based on the sensitivity, specificity, positive and negative predictive value, and positive and negative likelihood ratio. The serum was considered positive if the OD was greater than the cut-off value.

### Statistical analysis

The receiver operating characteristic (ROC) curve was plotted using the statistical software package spss 16.0 for Windows (SPSS). The true positive, false positive and area under the ROC curve (AUROC) was determined to illustrate the sensitivity versus specificity of the in-house IgM/IgG ELISA. The differences were considered to be statistically significant at *P*<0.05.

## Results

**Fig. 1.**
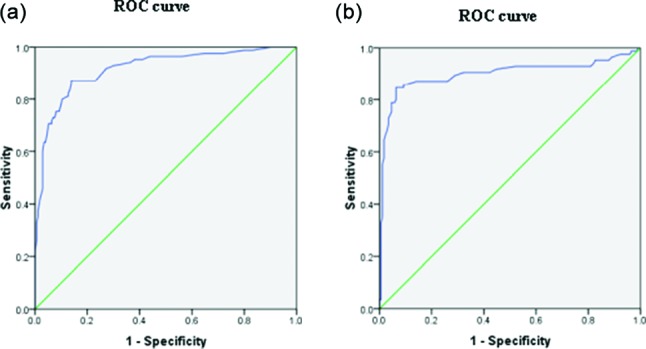
ROC curve showing the sensitivity and 1 – specificity for serodiagnosis of melioidosis using in-house IgM (a) and IgG (b) ELISAs.

In this study, comparison between the IgM and IgG ELISA showed a higher absorbance value of IgG compared to IgM in all the tested samples. Overall, the IgG ELISA performed better than IgM ELISA with higher sensitivity and specificity and both positive and negative predictive values of more than 80 %. The likelihood ratio for both assays did not differ to any great extent [13.32, 7.75 (LR+), 0.16, 0.26 (LR–)] (Table 1). The ROC analysis showed that the AUROC values for both IgM and IgG ELISA were 0.917 [95 % confidence interval (CI): 0.879, 0.955; *P*<0.05] and 0.899 (95 % CI: 0.847, 0.951; *P*<0.05), respectively (Fig. 1).

**Table 1.** Sensitivity, specificity, predictive values (positive, negative) and likelihood ratios (positive, negative) of in-house ELISAs at different cut-off values PPV, positive predictive value; NPV, negative predictive value; LR+, positive likelihood ratio; LR–, negative likelihood ratio.

Antibody	Cut-off value	Sensitivity (%)	Specificity (%)	PPV (%)	NPV (%)	LR+	LR–
IgM	0.25	85.88	86.13	75.26	91.98	6.19	0.16
	0.30	76.14	90.17	79.76	89.66	7.75	0.26
	0.35	72.94	93.06	83.78	87.03	10.52	0.29
	0.40	68.24	95.95	89.23	85.57	16.86	0.33
IgG	0.45	85.88	90.17	81.11	92.86	8.74	0.15
	0.50	84.71	93.64	86.75	92.57	13.32	0.16
	0.55	81.18	93.64	86.25	91.01	12.77	0.20
	0.60	77.65	95.38	89.19	89.67	16.79	0.23

Using the IgG ELISA at a cut-off value of 0.50, among the 85 culture-confirmed sera, 13 tested negative compared to 18 of IgM at a cut-off value of 0.30. Among the negative controls in the IgM ELISA, only eight healthy donors (108), three leptospirosis (20), one of each legionellosis (11), ricketssiosis (15), brucellosis (10) respectively, and three *B. cepacia* culture-positive (9), were found IgM positive. On the other hand, for the IgG ELISA, only five healthy donors (108), one from each leptospirosis (20), legionellosis (11) and brucellosis (10) plus two *B. cepacia* culture-positive (9), but none with ricketssiosis (15), were found to be IgG positive.

**Fig. 2.**
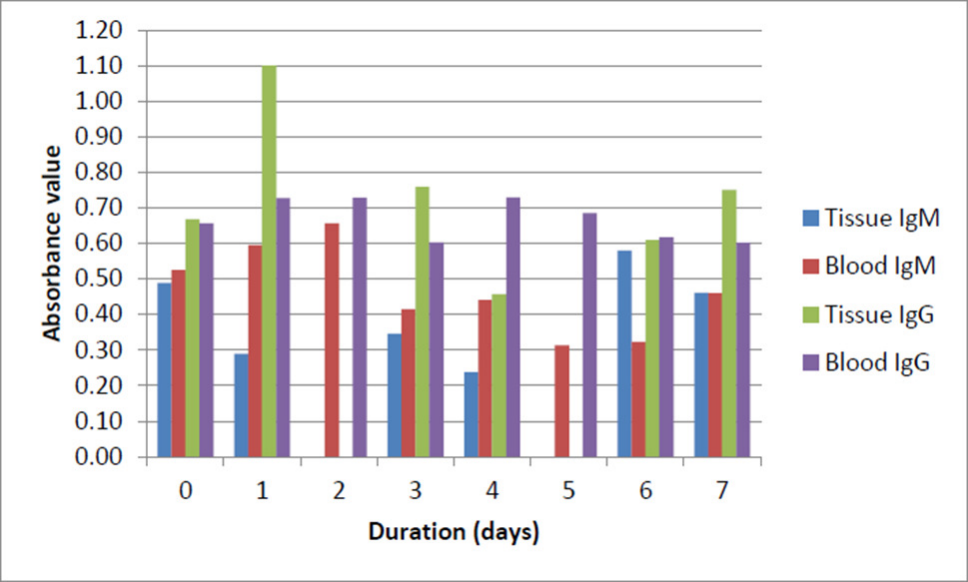
Comparison of the IgM and IgG level (absorbance value) between septicaemic and localized melioidosis patients. 'Duration' refers to the period between first blood sampling for culture (gold standard) and serology (IgM and IgG). Day 0: the date of blood sampling for culture and serology was the same. Day 7: the blood was drawn and sent for serology testing on the 7th day after the blood sampling for culture (day 1).

According to Fig. 2, out of 85 culture-positive sera, a total of 73 serum samples were taken within 7 days between blood sampling for culture and serology. There were 57 septicaemic and 16 localized infection cases used in the analysis. There were no cases of sampling at 2 and 5 days, which met our concern for localized blood samples. Overall, from our analysis, the IgG ELISA remained better than IgM for both localized and septicaemic infections from day 0 to day 7.

## Discussion

In this study, the comparison between in-house IgM and IgG ELISAs showed that IgG is a better indicator for early serodiagnosis of melioidosis than IgM with an overall higher sensitivity, specificity, and positive and negative predictive value and likelihood ratios. A suitable cut-off value is used to exclude background antibody levels to provide a sensitive and specific diagnosis of melioidosis [12–14]. Determination of the cut-off value is crucial in interpretation of the results. In our study, increasing or reducing the cut-off value led to false negative or false positive results. The range of cut-off values used in this study was derived from the mean (±sd) OD of the blood donors’ sera. When using the cut-off value determined, 0.30 for IgM and 0.50 for IgG, the IgG ELISA was able to discriminate between positive and negative sera with 84.71 % (sensitivity), 93.64 % (specificity), 86.75 % (PPV), 92.57 % (NPV), 13.32 (LR+) and 0.16 (LR–) compared to the IgM ELISA: 76.14 % (sensitivity), 90.17 % (specificity), 79.76 % (PPV), 89.66 % (NPV), 7.75 (LR+) and 0.26 (LR–). The high predictive values portray the accuracy in differentiating positive versus negative sera in diseased and non-diseased patients. The likelihood ratios were also calculated to further support the usefulness of the in-house IgG ELISA. The higher value of LR+ and the closer the value of LR– to 0, the greater value of the diagnostic test [12].

Furthermore, the ROC curve was also determined in this study. The ROC curve has been used extensively in diagnostic tests to revise the probability of disease in individual subjects [12]. The ROC curve was constructed using the data collected in this study. True positive refers to *B. pseudomallei* culture-positive sera (gold standard) and negative controls were the other *B. pseudomallei* culture-negative sera. Despite having a slightly lower AUROC value for IgG compared to IgM, the diagnostic potential of IgG is further supported when the IgM and IgG absorbance value was compared between first blood sampling for culture versus serology. The analysis showed that the IgG level in both localized and septicaemic infection was notably high compared to IgM from day 0 to day 7. Culture is recognized as the gold standard for the diagnosis of melioidois, but the incubation period for the growth of *B. pseudomallei* from clinical specimens is at least 3–5 days [3] and from our preliminary findings, prior to growth of the organism, we are able to identify the patient as serologically positive for melioidosis using the in-house IgG ELISA at a chosen cut-off value of 0.50.

In north-east Thailand, more than 80 % of children in rice-farming communities were found to have acquired antibodies against *B. pseudomallei* by the time they are 5 years old due to continuous exposure to the bacteria [7]. However, James and co-workers reported a different observation showing that in Darwin (Northern Territory, Australia), a melioidosis-endemic area, there was a low seroprevalence of *B. pseudomallei* among exposed healthy adults [15]. In this study, we compared the diagnostic potential of both IgM and IgG using an in-house ELISA method. The results showed that IgG is better than IgM in the early diagnosis of melioidosis, a different view from that of Ashdown [16], and is comparable and in agreement with recent reports that IgG is a better diagnostic indicator for melioidosis [3, 17–22]. Using the cut-off value of 0.50, the non-melioidosis sera were ruled out at a specificity of 93.64 % and a sensitivity of 84.71 %. Only 4.6 % of the healthy blood donors were noted as positive for IgG compared with 7.4 % for IgM. There were 85.94 and 80.95 % of the septicaemic and localized infections detected using the in-house IgG ELISA compared to 84.38 and 61.9 % for IgM, respectively, which is comparable with data from Ho *et al*. [21] who reported that there was little difference in IgG level between septicaemic and localized infections. However, we noted a slight difference with more septicaemic cases detected compared to using the in-house IgM ELISA [21]. Positive sera tested against several Gram-negative bacteria were included in the study to rule out the possibility of cross-reaction. The in-house IgG ELISA showed a low prevalence or no cross-reaction with other Gram-negative bacteria such as *Legionella pneumonia, Rickettsia* species, *Brucella* species and *Leptospira* species. Comparison with the near-neighbour species *Burkholderia cepacia* showed a possible cross-reactivity of 22 % (two out of nine) of the sera reacting towards the *B. pseudomallei* antigen, but the sample size is too low to draw definitive conclusions.

Besides being a rapid and inexpensive method, previous studies from Thailand reported that by using Bayesian latent-class models, ELISA was more accurate and favourable in the serodiagnosis of melioidosis [8, 9] as compared with the true sensitivity of the traditional culture method (gold standard), which was only 60 % [9]. The ELISA method is widely favoured because it is user-friendly, cost-effective and simple. For example, ELISA has been applied in numerous procedures to evaluate the diagnostic accuracy of different antigens in the serodiagnosis of melioidosis [23–27]. Although IHA is used widely for the serodiagnosis of melioidosis, there are numerous reports on its low sensitivity and specificity as well as the inability to differentiate stages of infection in endemic areas [5, 7, 28–30].

The use of IgG in ELISA is supported by numerous findings on its efficiency as compared to IgM [17–19, 22, 24, 27, 30, 31]. Therefore, IgG assessment will be helpful in the early diagnosis of melioidosis. Based on our analysis, the use of the in-house IgG ELISA method is appropriate and performs well. Our in-house IgG ELISA and the chosen cut-off value provides a reliable method for the serodiagnosis of melioidosis in Malaysia coupled with valid clinical presentations.

In this study, whole-cell antigen was used as the coating base for the in-house ELISA. Despite the discovery and report of numerous potential serodiagnostic markers for melioidosis, the results were variable. The identified targets such as BPSS1904, BPSL3130 [24], recombinant truncated flagellin [25], OmpA [26], TssD-5 [27], OPS [22] and HCP1 [32] showed sensitivity and specificity of 71–95 % and 88–98 %, respectively. However, details of the geographical location (endemic status), type of strain and evaluation with a large number of samples will be necessary to provide the most effective serodiagnostic markers for Malaysian local populations. Additional study is needed on the local *B. pseudomallei* isolates to identify target antigens with higher sensitivity. Further monitoring on the population's basal antibody titre is required to enhance the efficacy of the in-house IgG ELISA.

### Conclusion

The in-house ELISA method using IgG has a better diagnostic potential compared to IgM and is suitable for our local hospital setting for the early diagnosis of suspected melioidosis patients.
